# Exploring listening-related fatigue in children with and without hearing loss using self-report and parent-proxy measures

**DOI:** 10.3389/fped.2023.1127578

**Published:** 2023-02-28

**Authors:** Bethany Adams, Sally K. Thornton, Graham Naylor, Ruth V. Spriggs, Ian M. Wiggins, Padraig T. Kitterick

**Affiliations:** ^1^ National Institute for Health and Care Research (NIHR) Nottingham Biomedical Research Centre, Nottingham, United Kingdom; ^2^ Hearing Sciences, Mental Health and Clinical Neurosciences, School of Medicine, University of Nottingham, Nottingham, United Kingdom; ^3^ National Acoustic Laboratories, Australian Hearing Hub, Macquarie University, Sydney, NSW, Australia

**Keywords:** fatigue, children, unilateral hearing loss, self-report, hearing loss, quality of life

## Abstract

Children with hearing loss appear to experience greater fatigue than children with normal hearing (CNH). Listening-related fatigue is often associated with an increase in effortful listening or difficulty in listening situations. This has been observed in children with bilateral hearing loss (CBHL) and, more recently, in children with unilateral hearing loss (CUHL). Available tools for measuring fatigue in children include general fatigue questionnaires such as the child self-report and parent-proxy versions of the PedsQL^TM^-Multidimensional Fatigue Scale (MFS) and the PROMIS Fatigue Scale. Recently, the Vanderbilt Fatigue Scale (VFS-C: child self-report; VFS-*P*: parent-proxy report) was introduced with a specific focus on listening-related fatigue. The aims of this study were to compare fatigue levels experienced by CNH, CUHL and CBHL using both generic and listening-specific fatigue measures and compare outcomes from the child self-report and parent-proxy reports. Eighty children aged 6–16 years (32 CNH, 19 CUHL, 29 CBHL), and ninety-nine parents/guardians (39 parents to CNH, 23 parents to CUHL, 37 parents to CBHL), completed the above fatigue questionnaires online. Kruskal-Wallis H tests were performed to compare fatigue levels between the CNH, CUHL and CBHL. To determine the agreement between parent-proxy and child self-report measures, Bland-Altman 95% limits of agreement were performed. All child self-report fatigue measures indicated that CBHL experience greater fatigue than CNH. Only the listening-specific tool (VFS-C) was sufficiently able to show greater fatigue in CUHL than in CNH. Similarly, all parent-proxy measures of fatigue indicated that CBHL experience significantly greater fatigue than CNH. The VFS-*P* and the PROMIS Fatigue Parent-Proxy also showed greater fatigue in CUHL than in CNH. Agreement between the parent-proxy and child self-report measures were found within the PedsQL-MFS and the PROMIS Fatigue Scale. Our results suggest that CBHL experience greater levels of daily-life fatigue compared to CNH. CUHL also appear to experience more fatigue than CNH, and listening-specific measures of fatigue may be better able to detect this effect. Further research is needed to understand the bases of fatigue in these populations and to clarify whether fatigue experienced by CBHL and CUHL is comparable in nature and degree.

## 1. Introduction

There is no universally accepted definition of fatigue, though it is generally described as an overall feeling of tiredness, lack of energy or vigour, or decreased motivation (1). Fatigue often presents after insufficient sleep or increased mental or physical exertion, but usually resolves after rest or mental stillness.

It has been widely reported that both adults (2, 3) and children (4–9) with hearing loss experience an increase in daily life fatigue, compared to their normal hearing peers. Individuals with a hearing impairment may have to allocate more cognitive resources to effortful listening than those with normal hearing (10), which can lead to fatigue. This fatigue, being a result of listening, is referred to as ‘listening-related fatigue’. Children with hearing loss (CHL) have to exert greater listening effort than children with normal hearing (CNH), potentially leading to poorer sentence recognition (11) and lower processing speed (12) in listening tasks.

Moreover, children are more affected by unfavourable noise conditions than adults (13) as classroom teaching often takes place in a reverberant and noisy environment (14, 15), requiring considerable listening effort (16). The available literature suggests that for CHL it can be mentally exhausting spending an entire day listening to their teacher's speech, against excessive classroom noise levels. It is also more difficult for children with unilateral hearing loss (CUHL) to localise sounds, compared to CNH (17).

Children with hearing loss have reported fatigue in qualitative studies. Davis et al. (18) found that fatigue in CHL is expressed in many ways, such as difficulty concentrating, lack of motivation, and physical tiredness. Parents of CHL and clinicians who manage CHL have also noted that children with varying degrees of hearing loss experience fatigue, especially following sustained listening demands at school Bess et al. (4). Although the consequences of fatigue have not yet been measured in children with hearing loss, it has been reported that children with fatigue due to a chronic health disorder have poor academic performance, decreased motivation, increased distractibility, poorer social functioning, and more depressive symptoms (19–21). If such negative consequences also are present in CHL, then there is a pressing need for further elucidation.

Currently, fatigue is most often measured *via* self-report. This is often in the form of questionnaires but can also be *via* qualitative interviews. Often, subjective measures of fatigue are multidimensional, which capture health or activities commonly associated with fatigue, such as sleep, cognition, and social functioning (22).

The well-known and widely used Pediatric Quality of Life Multidimensional Fatigue Scale (PedsQL-MFS 23, 24); has been used in many studies to quantify fatigue in CHL (7, 9, 25, 26). This questionnaire assesses three domains of fatigue, namely *General Fatigue*, *Sleep/Rest Fatigue* and *Cognitive Fatigue*. A *Total Fatigue* score is calculated by summing the above three domains.

Hornsby et al. (2014) were the first to measure fatigue in CHL using self-report questionnaires. The PedsQL-MFS was used to measure fatigue in ten school aged children with bilateral hearing loss (CBHL), and ten aged-matched CNH. They found higher levels of fatigue in CHL compared to their CNH peers in all domains. These findings were confirmed in a larger study by the same group (9) in which parent-proxy reports of fatigue were also collected. Here, they compared fatigue ratings between 60 CBHL and 43 CNH. In this study, CBHL rated higher levels of fatigue across all domains, which was significant in the *Cognitive* and *Total* domains. They showed that parent ratings of fatigue were significantly different to both ratings by CHL and CNH in *Cognitive*, *Sleep/Rest* and *Total* domains of fatigue (9). In the parent-proxy report, parents often report higher than their children's self-report (higher scores represent lower fatigue), especially in the *Sleep/Rest* domain, suggesting that parents often underestimate their child's fatigue. However, it is important to note that this standardised tool was designed to measure fatigue in children with chronic health disorders, such as rheumatoid arthritis. It was not designed to measure fatigue in children with hearing loss, and so may not be considered the best tool to measure listening-related fatigue. A new, recently validated, measure for fatigue in CHL is the pediatric version of the Vanderbilt Fatigue Scale (VFS-Peds 27);. This stands out from the PedsQL-MFS, as it is the only tool which has been specifically designed to measure listening-related fatigue in children.

Until recently, researchers measuring fatigue in CHL did not include CUHL. Unilateral hearing loss (UHL) has historically been regarded as being a minor inconvenience, and CUHL are generally offered less support when compared to CBHL (28). However, increasing evidence has shown that having UHL can affect many aspects of a child's development in ways that can be considerably impactful; socially, educationally, and behaviourally (29–32).

A recent qualitative study provided supporting narrative evidence for the incidence of fatigue in CUHL (4). Focus groups with parents of children with UHL included the observation that “My daughter is exhausted most days after school or when she has to listen for a long time”. One audiologist observed that “Our kids with UHL are similar to children with mild to moderate hearing losses – they require auditory breaks throughout the day and struggle more academically than one would expect given their hearing loss.” Hornsby et al. (27) utilised the Vanderbilt Fatigue Scale (VFS-C: child self-report; VFS-*P*: parent-proxy report) to measure fatigue in CNH, CUHL, and CBHL, with self-report from the children and proxy reports from their parents. As expected, CBHL rated significantly more fatigue than CNH. Though CUHL rated a greater level of fatigue than CNH, this was not significant. Parents of CNH, CUHL and CBHL (PNH, PUHL, PBHL, respectively) completed the parent-proxy report (VFS-*P*). Interestingly, both PUHL and PBHL had very similar fatigue ratings, which were both significantly higher than PNH. PBHL or PUHL were approximately four times more likely to report that their child experiences moderate-to-severe fatigue than PNH (4). This exploratory study was the first to include CUHL and quantitatively show that they experience similar levels of fatigue to CBHL.

Two more recent studies have measured fatigue in CUHL using the highly validated PedsQL-MFS. Sindhar et al. (26) compared fatigue levels between CUHL and CBHL aged 5–18 years (mean age 10.7 years), children with normal hearing (CNH) children (obtained from 23) using both child self-report and parent-proxy versions of the PedsQL-MFS. In the child self-report version, CBHL reported significantly greater fatigue than CNH in the *Total*, *General* and *Cognitive* fatigue domains, but not the *Sleep/Rest* domain. Children with UHL also reported greater levels of fatigue than CNH, though there were no significant differences throughout domains. Conversely, in the parent-proxy reported fatigue scores, both PUHL and PBHL rated significantly greater levels of fatigue than CNH across all domains and there were no significant differences in parent-proxy reports between CUHL and CBHL. Similarly to reports from the VFS in Bess et al. (2020), parents of children with hearing loss who completed the parent-proxy PedsQL-MFS (26) reported much higher levels of fatigue than their children's self-report ratings, with a much greater distinction from the CNH. This contrasts to Hornsby et al. (9), where parents underestimated their children's fatigue levels. Carpenter et al. (25) compared fatigue levels in children aged 5–18 years (mean age 10.44 years) with unilateral sensorineural hearing loss (USNHL) and unilateral conductive hearing loss (UCHL) to CNH (obtained from 23), again using child self-report and parent-proxy versions of the PedsQL-MFS. They found that children with USNHL reported significantly greater levels of fatigue than CNH children across all fatigue domains other than the *Sleep/Rest* domain. Children with UCHL reported similar levels of fatigue to CNH children. Conversely, in the parent-proxy reported outcomes, parents of children with both USNHL and UCHL rated their children's fatigue at similar levels (though parents of children with USNHL rated the highest fatigue levels), both of which were significantly greater than the CNH scores, across all four fatigue domains. Carpenter et al. (25) suggested that the lack of perceived fatigue reported by children with UCHL, compared to children with USNHL, was because the former have better habilitation options, such as bone conducting hearing aids. Carpenter et al. also noted that in this study, children with USNHL had a greater degree of hearing loss than those in the UCHL group.

In most current literature, both the non-hearing specific PedsQL-MFS and the hearing specific VFS have been used to measure fatigue in CUHL and CBHL. These questionnaires have not yet been directly compared to each other to determine if they are in fact measuring the same type of fatigue. In this study, we compared fatigue levels between CNH, CUHL and CBHL using the PedsQL-MFS and the newly validated VFS. We also introduced the PROMIS Fatigue Short Form (33), a highly standardised measure of fatigue in children, that has not previously been used to quantify fatigue in children with hearing loss.

This study aims to explore the fatigue levels in CNH, CUHL and CBHL using generic and listening-specific fatigue questionnaires. We also aim to explore whether parent-proxy reporting is a reliable measure of fatigue in children with hearing loss.

## 2. Methods

Children aged 6–16 years with unilateral hearing loss (UHL), bilateral hearing loss (BHL) or normal hearing (NH), and their parents/guardians were invited to take part in an online questionnaire. The online questionnaire was advertised *via* NHS audiology clinics, schools, and online *via* social media. Parents/guardians who were interested in taking part were first invited to a webpage, which contained the participant information sheet and parent consent form. Parents/guardians were encouraged to read through this page with their children before signing up to take part. Once signed up, parents/guardians were sent two password protected study links *via* email. One for the parent/guardian to complete and one for the child to complete. For children aged 6–7, parents were advised to assist in the completion of questionnaires by reading out each individual statement and recording responses if necessary. For children aged 16 years, the study link was sent directly to them.

The study was created and hosted using Online Surveys (https://www.onlinesurveys.ac.uk/) and distributed electronically with personalised links *via* email. Before launch, the study was piloted by pediatric audiologists, clinical hearing scientists and their children (one CUHL and three CNH). For children aged 6–15 years, the study link first opened up to the child participant information sheet and consent and assent page. Parents were asked to electronically sign the consent form, and children were asked to sign the assent form before they could move onto the series of questionnaires. Children aged 16 were also presented with a participant information sheet and asked to sign the consent form before accessing the questionnaires.

### 2.1. Inclusion criteria

Children were eligible if they were aged 6–16 years with a permanent unilateral or bilateral hearing loss or normal hearing. Hearing loss was confirmed *via* parental self-report of diagnosis from a clinical audiologist. The study was entirely virtual, so formal hearing assessments were not possible. The following eligibility criteria were applied:

#### 2.1.1. Children

• Aged 6–16
• Has received a diagnosis of permanent unilateral hearing loss or permanent bilateral hearing loss
• Has normal or corrected-to-normal vision confirmed *via* parental self-report
• Uses English in home environment or at school
• Able and willing to give informed consent (16 years or older), or to give assent together with consent from a parent/guardian
• Has access to a computer/smart phone or tablet with internet.

#### 2.1.2. Parents/guardians

• A parent/guardian to a child who meets the above criteria
• Able to speak fluent English
• Has access to a computer with internet
• Able and willing to give informed consent

### 2.2. Study format

**Figure 1:**
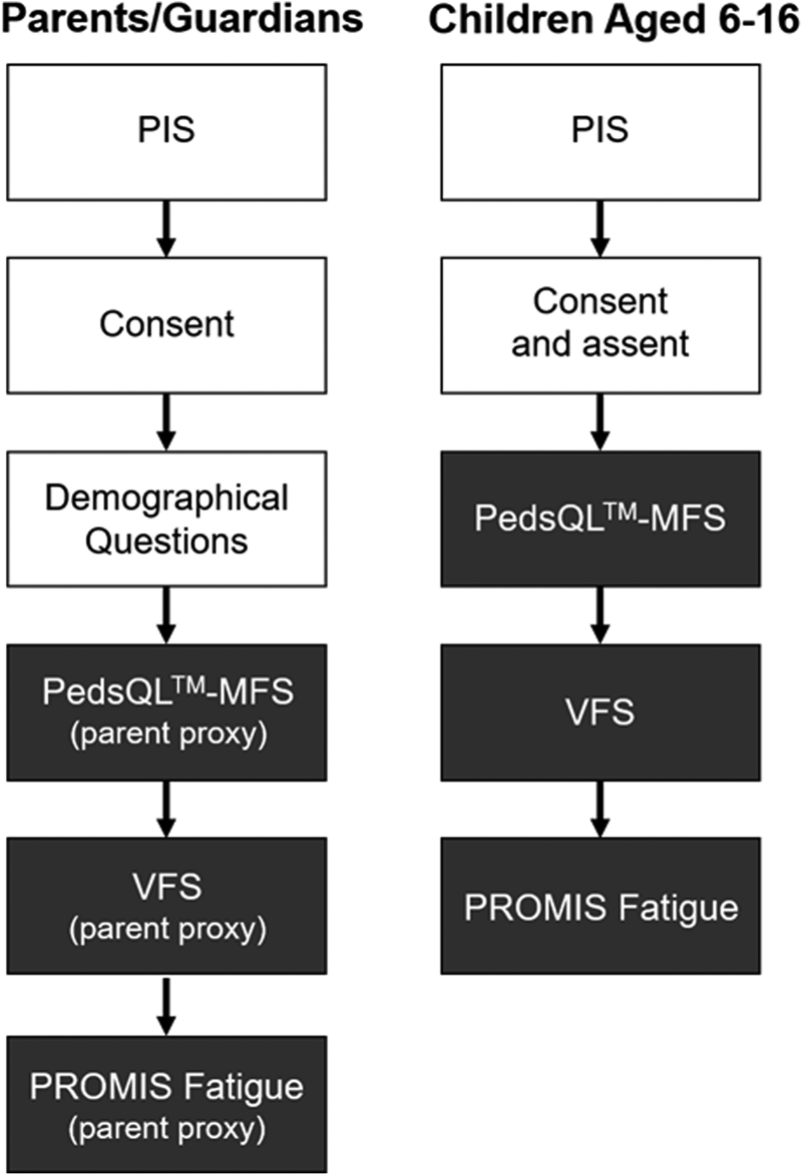
Flowchart of study format for parent/guardian and child participants. For child self-report section, parent consented for children aged 6-15 years old, and children assented. Children who were aged 16 provided their own consent. PIS = Patient Information Sheet. Shaded boxes are fatigue questionnaires.

The study format is outlined in Figure 1.

### 2.3. Recruitment

Participants were recruited through advertisement on online forums, in schools, and in audiology clinics.

### 2.4. Ethics

This study was approved by the Health Research Authority (HRA), Wales Research Ethics Committee (20/WA/0233) and the Nottingham Research Committee (52–0720).

### 2.5. Questionnaires

#### 2.5.1. PedsQL-MFS

The Pediatric Quality of Life Inventory Multidimensional Fatigue Scale (PedsQL-MFS; 23, 24) is a standardised self-report instrument designed to measure fatigue in paediatric patients. It consists of three separate fatigue domains: *General Fatigue* (6 items), *Sleep/Rest Fatigue* (6 items), and *Cognitive Fatigue* (6 items), and also gives a score of *Total Fatigue*. It includes both child and parent-proxy reports, allowing both perspectives. The PedsQL-MFS has been used by children with a wide variety of health conditions, including cancer (24) and rheumatoid arthritis, and has been shown to have good internal consistency, reliability and validity (23). For ease of use in younger children (aged 5–7), the PedsQL-MFS has simplified terminology, and answers are anchored to a happy/sad faces scale (23).

#### 2.5.2. Vanderbilt fatigue scale

The Vanderbilt Fatigue Scale (VFS; 4, 27) is a self-report tool designed to measure listening-related fatigue in children. All items in the VFS are directly related to hearing or listening, for example “My brain gets tired after listening all day” (27). The VFS includes three versions: child self-report (VFS-C), parent-proxy (VFS-*P*) and teacher-proxy (VFS-T).

#### 2.5.3. PROMIS paediatric fatigue short form

The Patient-Reported Outcomes Measurement Information System (PROMIS) paediatric self-report (33) and parent-proxy (34) fatigue short-forms (v10a) are validated questionnaires used to assess fatigue in multiple populations. An example item from this short form includes “I was too tired to enjoy the things I like to do”. Although these tools have not yet been used to measure fatigue in children with hearing loss, they have been shown to have a high content validity (35).

### 2.6. Scoring and statistics

The PedsQL-MFS responses were summed to produce a score for each domain (*General Fatigue*, *Sleep/Rest Fatigue* and *Cognitive Fatigue*). These scores were then combined to produce the *Total Fatigue* score. A lower score indicates a greater level of fatigue. The VFS was scored using Item Response Theory (IRT) using R Studio, as outlined by Hornsby et al. (27). Higher IRT scores indicated a greater level of fatigue. The PROMIS Fatigue Short Form raw scores were converted to T-Scores [https://www.assessmentcenter.net/]. T Scores are standard scores with a mean of 50 and a standard deviation of 10 in a reference population (36). Higher T Scores indicate a greater level of fatigue, and a score above 50 indicates greater fatigue than the population average.

Descriptive statistical analysis and normality tests were conducted using IBM SPSS Statistics for Windows, version 28.0 (IBM Corp., Armonk, NY). Data were not normally distributed. Normality of response measures was assessed visually with histograms and with the Shapiro-Wilk test. Kruskal-Wallis H tests, with Mann-Whitney post-hoc tests, were performed to compare fatigue levels between the CNH, CUHL and CBHL.

To determine the agreement between parent-proxy and child self-report measures of fatigue, Bland-Altman limits of agreement (37) were performed with exact 95% CI for the limits of agreement, and supplemented by Pearson correlation coefficients. Agreement calculations between the VFS-*P* and VFS-C were not possible due to different outcomes between parents and children (VFS-C gives one *Total Fatigue* score, whereas VFS-*P* gives separate scores of Mental Fatigue and Physical Fatigue).

## 3. Results

The study was open between August 2020 and September 2021. Ninety-nine parents/guardians completed the study, out of which 37 were parents to CBHL, 23 were parents to CUHL and 39 were parents to children with NH. Eighty children between the ages of 6 to 16 completed the questionnaires (mean age = 10.25; SD = 3.02), of which 29 children had BHL, 19 had UHL and 32 had NH. In 77 cases, both the parent and child completed the questionnaire (26 BHL, 19 UHL and 32 NH). Participant demographics can be found in Table 1.

**Table 1:** Demographics include 99 cases where the parent filled out the questionnaires. HA; hearing aids, CI; cochlear implants.

	Normal Hearing *N* = 39	Unilateral Hearing Loss *N* = 23	Bilateral Hearing Loss *N* = 37
***Gender of child***			
* Male*	**25**	**11**	**21**
* Female*	**14**	**12**	**16**
***Age of child***			
* 6-7*	5	6	11
* 8-12*	19	13	18
* 13-15*	13	4	5
* 16*	2	0	3
***Hearing Status***		**23**	**37**
* Permanent Hearing Loss*		**21**	**33**
* Fluctuating Hearing Loss*		**2**	**4**
***Hearing Devices***			
* HA*	0	**14**	**23**
* CI*	0	**1**	**11**
* FM System*	0	**3**	**22**

### 3.1. PedsQL-MFS

**Figure 2:**
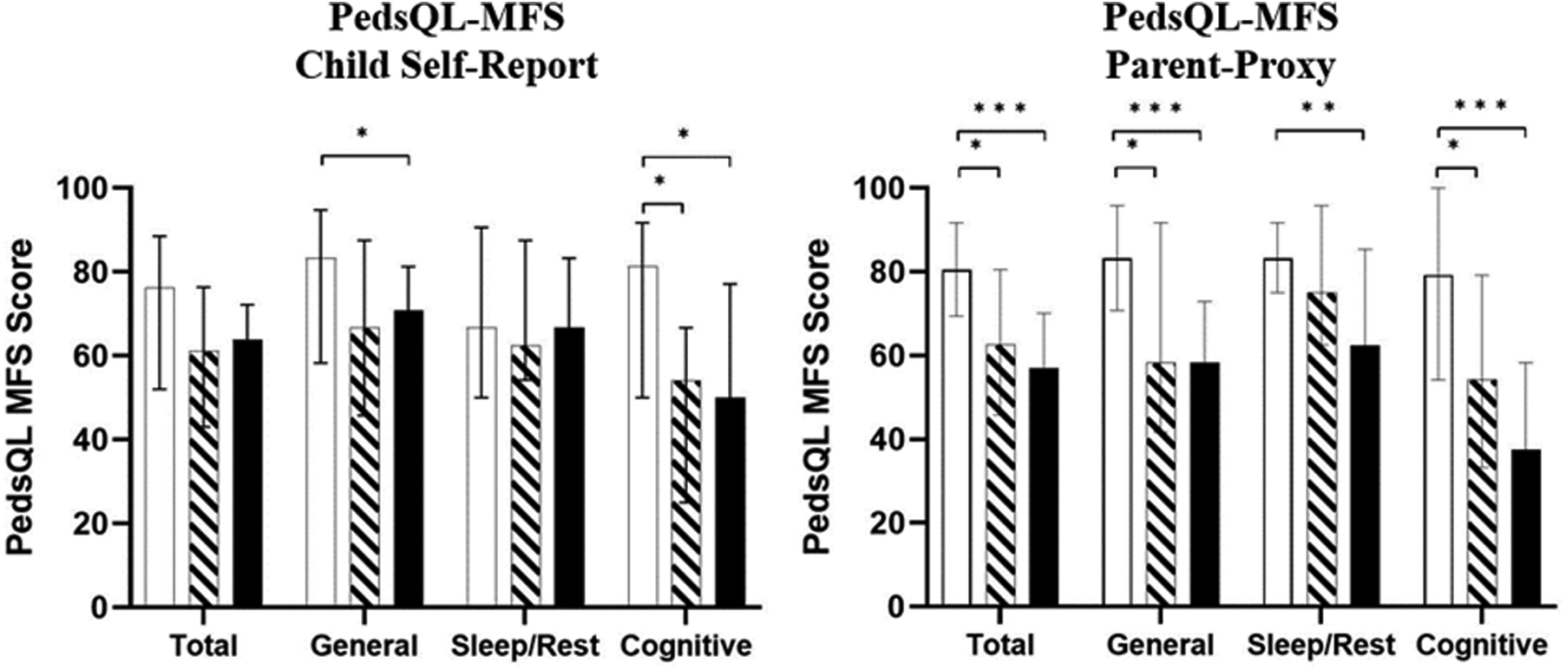
Median pediatric quality of life inventory multidimensional fatigue scale (pedsQL-MFS) scores for child self-report (left) and parent-proxy (right) respondents. Error bars shown represent medians ± IQRs. Lower values indicate greater fatigue. CNH; white bars, CUHL; oblique stripes, CBHL; black bars respectively. Asterisks show significant differences between groups (*p < 0.05, **p < 0.01, ***p < 0.001).

In the PedsQL-MFS, lower scores indicate a greater level of fatigue. Median scores, interquartile ranges (IQR), and minimum and maximum values for the child self-reported and parent-proxy PedsQL-MFS are reported in Table 2. Figure 2 displays the child self-reported medians and IQRs for CNH, CUHL and CBHL and parent-proxy reported means for CNH, CUHL and CBHL. Lower scores on the PedsQL-MFS indicate a higher level of fatigue.

**Table 2:** Medians (interquartile range) and [min, max] of child self-report and parent-proxy report scores from the pedsQL-MFS.

	NH	UHL	BHL
Child Self-Report	*N = 32*	*N = 19*	*N = 29*
Total	76.4 (36.5) [20.8, 100]	61.1 (33.3) [5.6, 98.6]	63.9 (23.6) [8.3, 91.7]
General	83.3 (36.5) [8.3, 100]	66.7 (41.7) [0.0, 100]	70.8 (35.4) [8.3, 95.8]
Sleep/Rest	66.7 (40.6) [25.0, 100]	62.5 (33.3) [0.0, 100]	66.7 (37.5) [16.7, 91.7]
Cognitive	81.3 (41.7) [0.0, 100]	54.2 (41.7) [0.0, 95.8]	50.00 (43.8) [0.0, 95.8]
**Parent-Proxy Report**	*N = 39*	*N = 23*	*N = 37*
Total	80.56 (2.2) [43.0, 100]	62.5 (34.7) [9.7, 97.2]	56.9 (38.9) [8.3, 98.6]
General	83.3 (25.0) [54.2, 100]	58.3 (50.0) [12.5, 100]	58.3 (41.7) [0.0, 95.3]
Sleep/Rest	83.3 (16.7) [37.5, 100]	75.0 (33.3) [8.3, 100]	62.5 (39.6) [8.3, 100]
Cognitive	79.2 (45.8) [0.0, 100]	54.2 (45.8) [0.0, 100]	37.5 (50.0) [0.0, 100]

CUHL and CBHL reported greater levels of fatigue than CNH across all domains. CUHL reported similar levels of fatigue to CBHL. A Kruskal-Wallis test was conducted to determine if there were differences in fatigue scores between groups (NH, UHL and BHL). No significant differences in fatigue scores were found in the *Total* (H(2) = 5.235, *p* = 0.07) or *Sleep/Rest* (H(2) = 0.688, *p* = 0.709) domains. Significant differences in fatigue scores between groups were found within the *General* (H(2) = 7.591, *p* = 0.022) and *Cognitive* (H(2) = 8.141, *p* = 0.017) domains. Subsequently, Mann-Whitney pairwise comparisons were performed with Bonferroni correction for multiple comparisons in the *General* and *Cognitive* fatigue domains. This *post hoc* analysis revealed statistically significant differences between CBHL and CNH in the *General* and *Cognitive* domains. CBHL reported statistically significantly greater levels of fatigue than CNH in the *General* (*p* = 0.031), and borderline significance for *Cognitive* (*p* = 0.05), domains of fatigue. CUHL reported significantly greater levels of fatigue than CNH in the *Cognitive* domain only, and this difference was marginally significant (*p* = 0.047).

Both groups of parents (CUHL and CBHL) reported greater fatigue for their children (indicated by lower PedsQL-MFS scores) than parents of CNH across all domains of fatigue (Table 2; Figure 2).

In the parent-proxy PedsQL-MFS, significant differences between child groups were found in all domains (*General*: H(2) = 23.926, *p* < 0.001; Sleep/Rest: H(2) = 10.508, *p* = 0.005; *Cognitive*: H(2) = 23.008, *p* < 0.001; *Total* H(2) = 23.828, *p* < 0.001). Parents of CBHL reported significantly greater levels of fatigue than CNH in the *Total* (*p* < 0.001), *General* (*p* < 0.001), *Sleep/Rest* (*p* = 0.004) and *Cognitive* (*p* < 0.001) domains. Additionally, parents of CUHL also rated significantly greater levels of fatigue than CNH across the *Total* (*p* = 0.017), *General* (*p* = 0.012) and *Cognitive* (*p* = 0.025) domains. There were no significant differences in parent-proxy ratings of fatigue between CUHL and CBHL.

#### 3.1.1. Parent-Proxy vs. Child self-report

For parent-proxy vs. child self-report comparisons, *N* = 77 for both groups. There were no significant differences in scores between child self-report and parent-proxy reports in the *Total* and *General* domains of the PedsQL-MFS. However, score differences between child self-report and parent-proxy reports of the *Sleep/Rest* and *Cognitive* domains were significant.

##### 3.1.1.1. Total

Agreement by Bland-Altman plots are shown in Supplementary Figure S1A with a mean difference of PedsQL-MFS *Total* score of −5.63 to 2.13; the limits of agreement were −35.21 to 31.71. The interclass correlation coefficient was 0.847 (95% CI: 0.759–0.903, *p* < 0.01). Regression analysis between the score means and the difference between scores were not significant (-0.52, *p* = 0.596), meaning there is no proportional bias.

##### 3.1.1.2. General

Agreement by Bland-Altman plots are shown in Supplementary Figure S1B with a mean difference of PedsQL-MFS *General* score of −4.61 to 5.26; the limits of agreement were −42.21 to 42.96. The interclass correlation coefficient was 0.785 (95% CI: 0.662–0.863, *p* < 0.001). Regression analysis between the score means and the difference between scores were not significant (-0.007, *p* = 0.949), meaning there is no proportional bias.

##### 3.1.1.3. Sleep/rest

Parent-proxy and child self-report PedsQL-MFS *Sleep/Rest* scores were significantly different (t(76) = −4.394, *p* < 0.001), so no Bland-Altman plot could be created, and proportional bias cannot be assessed. The interclass correlation coefficient was 0.845 (95% CI: 0.756–0.902, *p* < 0.001).

##### 3.1.1.4. Cognitive

Parent-proxy and child self-report PedsQL-MFS *Cognitive* scores were significantly different (t(76) = 6.858, *p* < 0.001), so therefore no Bland-Altman plot could be created, and proportional bias cannot be assessed. The interclass correlation coefficient was −0.389 (95% CI: −1.186 - −0.117, *p* = 0.923).

### 3.2. The VFS

**Figure 3:**
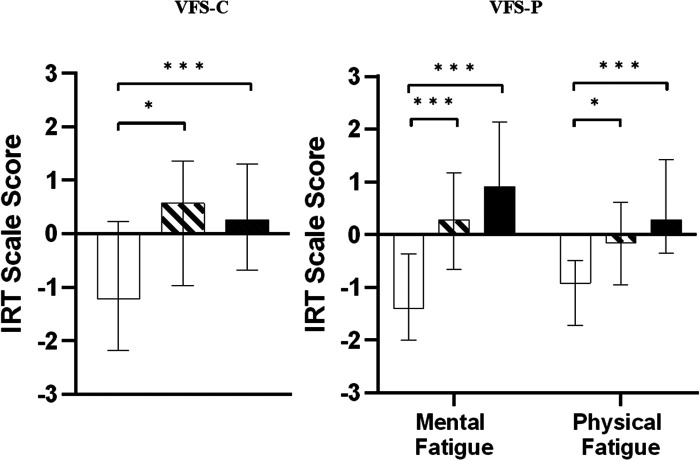
Median item response theory (IRT) scale scores for the VFS-children (left) and VFS-parent (right). Greater values indicate a greater level of fatigue. Bars and error bars shown represent medians ± IQRs. CNH; white bars, CUHL; oblique stripes, CBHL; black bars respectively. Asterisks show significant differences between groups (*p < 0.05, **p < 0.01, ***p < 0.001).

Median item response theory (IRT) scale scores, IQRs and minimum-maximum scores are shown in Table 3. Figure 3 displays the median IRT scores with their respective IQRs for CNH, CUHL and CBHL for both child self-report and parent-proxy report from the VFS. A higher score indicates a greater level of fatigue.

**Table 3:** Medians (interquartile range) and [min, max] of child self-report and parent-proxy report scores from the VFS.

	NH	UHL	BHL
**VFS-C**	*N* = 32	*N* = 19	*N* = 29
Total Fatigue	−1.2 (2.4) [−2.5, 2.7]	0.6 (2.3) [−2.5, 2.7]	0.3 (2.0) [−1.7, 2.7]
**VFS-*P***	*N* = 39	*N* = 23	*N* = 37
Mental Fatigue	−1.4 (1.6) [−2.0, 0. 7]	0.3 (1.8) [−2.0, 2.3]	0.9 (1.8) [−1.7, 2.4]
Physical Fatigue	−0.9 (1.2) [−2.3, 0.8]	−0.2 (1.6) [−2.3, 2.0]	0.3 (1.8) [−2.3, 2.0]

In the VFS-C (child self-report), both hearing loss groups (CUHL and CBHL) reported greater levels of fatigue than CNH. Fatigue scores were statistically significantly different between CNH, CUHL and CBHL (H(2) = 13.892, *p* < 0.001). Pairwise comparisons with Bonferroni corrections found CBHL experience significantly greater fatigue than CNH (*p* = 0.001). CUHL also experienced significantly greater fatigue than CNH (*p* = 0.029). There were no significant differences in fatigue levels between CUHL and CBHL in the VFS-C, though CUHL reported a higher level of fatigue than CBHL.

The VFS-*P* (parent-proxy) splits fatigue into two subdomains, mental fatigue and physical fatigue. Fatigue scores were statistically significantly different between CNH, CUHL and CBHL in both the Mental fatigue (H(2) = 48.644, *p* < 0.001) and Physical fatigue (H(2) = 30.364, *p* < 0.001) subdomains. In the VFS-*P*, again both CUHL and CBHL reported significantly greater levels of fatigue than CNH in the Mental fatigue domain (CBHL-CNH, *p* < 0.001; CUHL-CNH, *p* < 0.001) and the Physical fatigue domain (CBHL-CNH, *p* < 0.001; CUHL-CNH, *p* = 0.011). Parents of CBHL reported the greatest level of fatigue (for their children) compared to parent ratings for CUHL and CNH in both the Mental and Physical fatigue domains, whilst fatigue levels rated by parents of CUHL fall between ratings by parents of CBHL and parents of CNH. There were, however, no statistically significant differences between fatigue levels for CUHL and CBHL as rated by parents.

### 3.3. PROMIS fatigue short form

**Figure 4:**
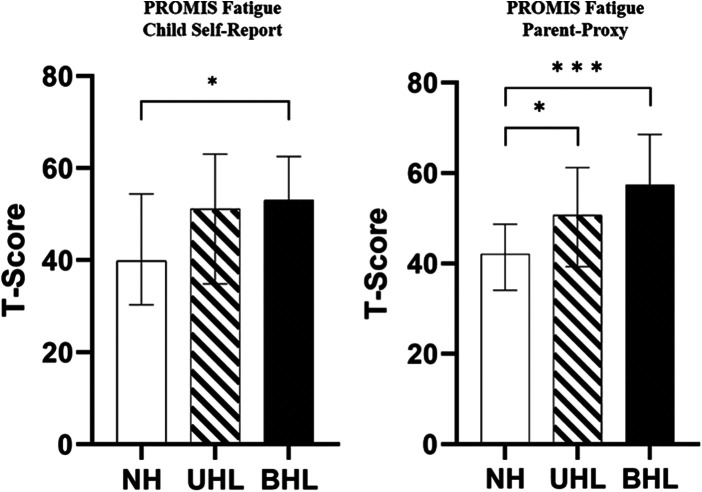
Median PROMIS fatigue short form 10a T-scores for the child self-report (left) and parent-proxy (right) respondents. Higher values indicate greater fatigue. Bars and error bars shown represent medians ± IQRs. CNH; white bars, CUHL; oblique stripes, CBHL; black bars respectively. Asterisks show significant differences between groups (*p < 0.05, **p < 0.01, ***p < 0.001).

T-scores for the child self-report and parent-proxy PROMIS Fatigue Short Form are shown in Table 4. Figure 4 displays the median T-scores with their respective IQRs for CNH, CUHL and CBHL for both child self-report and parent-proxy report from the PROMIS Fatigue Short Form. In the child self-report, there was a statistically significant difference between fatigue score and hearing loss groups (CNH, CUHL and CBHL; H(2) = 8.33, *p* = 0.016). Both CUHL and CBHL scored higher than CNH, though only CBHL scored a significantly greater level of fatigue than CNH (*p* = 0.017).

**Table 4:** Medians (interquartile range) and [min, max] of child self-report and parent-proxy report from the PROMIS fatigue short form.

	NH	UHL	BHL
**PROMIS Fatigue Child Self-Report**	*N* = 32	*N* = 19	*N* = 29
T Score	39.8 (24.1) [30.3, 76.3]	51.1 (28.2) [30.3, 81.5]	53.0 (18.7) [30.3, 80.0]
**PROMIS Fatigue Parent-proxy**	*N* = 39	*N* = 23	*N* = 37
T Score	42.1 (14.6) [34.1, 61.0]	50.7 (21.9) [34.1, 75.2]	57.4 (18.2) [34.1, 80.8]

In the parent-proxy report, fatigue scores were statistically significantly different between groups (H(2) = 28.325, *p* < 0.001). Parents of CBHL and parents of CUHL scored significantly greater fatigue scores than parents of CNH (CBHL-CNH, *p* < 0.001; CUHL-CNH, *p* = 0.015).

#### 3.3.1. Parent-Proxy vs. Child self-report

Agreement by Bland-Altman plots are shown in Supplementary Figure S1C with a mean difference of PROMIS Fatigue score of −1.72 (95% CI: −4.16 – 0.72); the limits of agreement were −22.78 (95% CI: −18.60 – −26.96) to 19.34 (95% CI: 15.15–23.53). The interclass correlation coefficient was 0.813 (95% CI: 0.705–0.881, *p* < 0.001). Regression analysis between the score means and the difference between scores was not significant (0.70, *p* = 0.482), meaning there is no proportional bias.

## 4. Discussion

In this study, we compared fatigue levels experienced by children with NH, UHL and BHL using three separate measures of fatigue to: a) understand which questionnaires were sufficiently able to discriminate levels of fatigue between children with different types of hearing loss; and b) help us to understand whether parent-proxy reports of fatigue using these measures were good alternative measures to child self-reports. We found that both the general (PedQL-MFS and PROMIS Fatigue) and listening-specific (VFS) fatigue measures were able to detect that CBHL experienced significantly greater levels of fatigue than CNH, in both child self-report and parent-proxy measures. Out of the three child self-report fatigue measures used, only the listening-specific VFS had the ability to discriminate between fatigue levels in CUHL and CNH. Further, parents of CUHL reported significantly greater fatigue compared to parents of CNH in both the VFS-*P* and the PROMIS Fatigue Parent-Proxy measures, as well as the *Total*, *General* and *Cognitive* domains of the PedsQL-MFS.

The PedsQL-MFS has been used the most extensively to measure fatigue in CHL (7, 9, 25, 26). In our data, both CUHL and CBHL and their parents rated significantly greater *Cognitive* fatigue than CNH and their parents. These data most closely relate to the data presented by the listening-related VFS, and are suggestive of an increase in cognitive load due to listening effort. Marsella et al. (38) demonstrated an increase in cognitive load in children with asymmetric hearing loss by measuring alpha power in the parietal cortex using EEG. They found significantly higher parietal alpha power levels in noisy conditions compared to quiet listening conditions. These increased cognitive demands in children with hearing loss led to an increase in cognitive fatigue. In a study by Brännström et al. (39), native and non-native language speaking children underwent listening comprehension tests whilst pupil dilation was measured using pupillometry. They found pupil dilation to be greater in poorer listening conditions, indicating an increased listening effort. They also found that baseline pupil size decreased over the listening comprehension trials, indicating listening-related fatigue. This study demonstrates the dynamics between listening effort and cognitive load during difficult listening situations. However, Alhanbali et al. (3) assessed fatigue and effort in hearing-impaired participants using the Fatigue Assessment Scale and Effort Assessment Scale. There were no significant differences between effort and fatigue scores, and the measures were only weakly correlated. These findings could suggest that listening effort is not the only predictor of fatigue and that there are potentially many other factors that could give rise to fatigue in hearing-impaired individuals. The development of fatigue may be produced by the lack of motivation to sustain effort within a particular task (40, 41). Throughout most domains of the PedsQL-MFS, parents of CUHL and CBHL rated lower levels of fatigue than their children. This is similar to the effect seen in Hornsby et al. (9), where parents rated their children as having significantly less fatigue than child self-report in the *Sleep/Rest*, *Cognitive* and *Total* domains. In our findings, though there was agreement between parent and child reports within the *Total* and *General Fatigue* domains, no agreement was found within the *Cognitive* and *Sleep/Rest* domains. This differs from Sindhar et al. (26) and Carpenter et al. (25) where no significant differences between parent proxy and child reports were found in any fatigue domains. There is little consistency in literature concerning the agreement between parent-proxy and child self-report outcomes. Ultimately, this is most likely due to individual relationships between parents and their children. It could also be due to different circumstances under which participants completed the questionnaires. The lack of consistency between parent-proxy and child self-report comparisons suggests that, if able to, children should complete their own fatigue self-report measures if required clinically.

This study is the first to compare fatigue levels in children with differing levels of hearing loss within the United Kingdom, the former studies were conducted within the United States. Parent child relationships across different cultures vary greatly (42) and understanding of fatigue may vary between nations. Furthermore, this study took place online as it took place during the Covid-19 pandemic, whilst Sindhar et al. (26), Carpenter et al. (25) and Hornsby et al. (9) administered the questionnaire in person. Online and in-person questionnaires both have their benefits and risks. Participants completing a survey online may feel more comfortable and free, as they are safe behind an extra layer of anonymity. However, completing a questionnaire online also gives the freedom to complete it at any time of the day, rather than a booked session that would usually be within working hours.

Although the PROMIS Fatigue Short form has not previously been used to measure fatigue in CHL, and was initially designed to measure fatigue in children with or without chronic pain (43), it was able to detect more fatigue in children with hearing loss compared to children with normal hearing in our sample. Though this questionnaire is unidimensional, results show similarity to scores within the PedsQL-MFS. The PROMIS showed more consistency between child self-report ratings and parent-proxy ratings than the multidimensional PedsQL-MFS.

Out of the three fatigue questionnaires, unsurprisingly the results show that the VFS was the most sensitive measure, as it was able to detect significantly greater fatigue in both CUHL and CBHL, compared to CNH, in both the child self-report and parent-proxy tools. This tool is an important step in helping us to understand fatigue in this population. This is a newly developed and newly validated tool, and is currently the only measure available that was designed to specifically assess listening-related fatigue in CHL (4, 27).

Until recently, the effects of UHL in children have been understudied, and assumed to be minimal (44). For example, children with UHL in the United Kingdom are not routinely funded to have a remote microphone system (RMS), a wireless microphone system that transmits sound from a talker to the receiver's ear (28), even though use of RMS has been shown to improve performance in sustained auditory attention ability (45). Unilateral hearing loss does, in fact, have many consequences (46); CUHL perform worse in localisation tasks compared to CNH (17, 47), and have poorer speech and language comprehension, reduced word recognition (17) and lower IQ scores (48, 49), to name a few. In this study, we found no significant differences in fatigue scores between CUHL and CBHL in child self-report questionnaires. Our data illustrate the need for continued research into the effects and impacts of unilateral hearing loss on children, to support the development of interventions to reduce this impact and the recognition of the needs of CUHL in policy and service provision.

### 4.1. Limitations

This study was open during national lockdowns due to the COVID-19 pandemic. Most children who took part in the study took part in school lessons from home, separated from their usual routines, whilst some children carried on going into school. Changes in their usual routines could have greatly affected responses in questionnaires. For example, children who still attended school may have been subjected to louder environments than usual due to mixing of age groups in classrooms, therefore exerting more listening effort. In classrooms, a predictor of fatigue, measured using the PedsQL-MFS, was found to be perceived listening difficulty (50). Children from single-child households may have had a quieter, more peaceful, work environment compared to children living in multiple-child households. As the study took place online, participants were not able to ask for clarifications from the researcher, as they would be able to do if they were completing questionnaires in person. It is also possible that children and parents completed the questionnaires together, causing the results to lack independence. Furthermore, the study was open for a year, and so fatigue levels may have differed depending on seasonal effects such as time spent outdoors.

Using a generic rather than a disease-specific outcome measure may be common in the clinical environment, but not always advisable for children with hearing loss. However, for CBHL the generic fatigue questionnaire measures were found to be acceptable and overall, produced quite similar findings to the outcomes from hearing specific questionnaires. Unfortunately, where CUHL measures are concerned then only the VFS-C was able to discriminate between the fatigue levels for CUHL and CNH.

Fatigue is complex and definitions and descriptions of fatigue can vary greatly, often depending on their source. For example, physical fatigue, defined as “the reduced desire to take part in physically demanding tasks, or reduced ability to maintain optimal performance” (51), is different from mental fatigue, described as a “reduced ability or desire to perform tasks that require concentration, attention, clear thinking and memory” (52). The broadness of fatigue as a construct renders measurement difficult, so it is therefore prudent to ascertain which aspects of fatigue demand most focus. Multidimensional fatigue scales, such as the 18-item PedsQL-MFS, can be advantageous as they capture a plurality of distinct domains in which fatigue may manifest, therefore providing a more nuanced description of impact than unidimensional scales. A negative consequence of this is that they are time-consuming and individual questions are not always relevant to the respondent. This is particularly prominent in questionnaires that are designed for the general population, rather than for specific cohorts. By contrast, the VFS-Peds is a 10-item questionnaire, which focuses on one source of fatigue (listening), but integrates several outcome domains in one score. For children with hearing loss, the VFS-Peds is more relevant and less time consuming than the PedsQL-MFS.

## 5. Conclusion

This study has measured fatigue in children with normal hearing (CNH), children with unilateral hearing loss (CUHL) and children with bilateral hearing loss (CBHL) using three separate tools for measuring fatigue. Our results suggest that both CUHL and CBHL experience more fatigue than their normal hearing peers, and that the listening-specific VFS may be better able to detect fatigue in CUHL. Research is needed to determine the potential differences or similarities in the experiences of CUHL, compared to CBHL, in order to understand the nature of fatigue in these populations so that we can ultimately find ways to improve their quality of life and reduce their fatigue.

## Data Availability

The raw data supporting the conclusions of this article will be made available by the authors, without undue reservation.
