# Supplementary material for: Exploring listening-related fatigue in children with and without hearing loss using self-report and parent-proxy measures
Source: Front Pediatr. 2023 Feb 28;11:1127578. doi: 10.3389/fped.2023.1127578 (PMC10011639; doi:10.3389/fped.2023.1127578)
Supplement: Supplementary file 1 [file Image1.pdf]

Supplementary Material

Exploring listening-related fatigue in children with and without hearing loss using self-report and parent-proxy measures.

Bethany Adams[1,2*], Sally Thornton[1,2], Graham Naylor[1,2], Ruth V. Spriggs [1,2], Ian Wiggins[1,2†], Padraig Kitterick[3†]

[1] National Institute for Health and Care Research (NIHR) Nottingham Biomedical Research Centre

[2] Hearing Sciences, Mental Health and Clinical Neurosciences, School of Medicine, University of Nottingham

[3] National Acoustic Laboratories, Australian Hearing Hub, Macquarie University NSW 2109, Sydney, Australia

[*] **Correspondence:**

Bethany Adams

Bethany.Adams@nottingham.ac.uk

† These authors contributed equally to this work and share last authorship

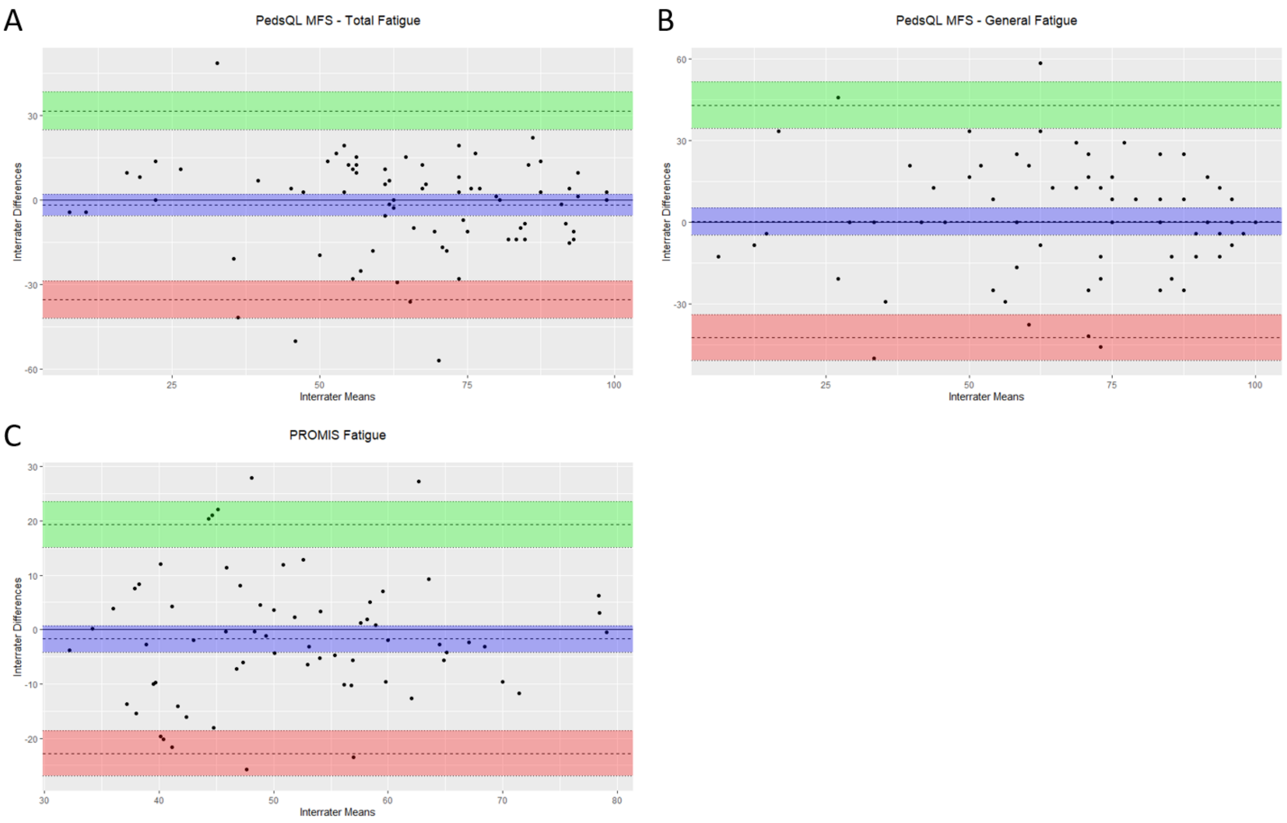

Supplementary Figure 1. These Bland-Altman plots illustrate the agreement between parent and child scores for the (A) PedsQL-MFS Total, (B) PedsQL-MFS General and (C) PROMIS Fatigue questionnaires. Lines are plotted indicating the limits of agreement (0±1.96 S.D)
